# Implementing AI-Driven Bed Sensors: Perspectives from Interdisciplinary Teams in Geriatric Care

**DOI:** 10.3390/s24216803

**Published:** 2024-10-23

**Authors:** Cromwell G. Acosta, Yayan Ye, Karen Lok Yi Wong, Yong Zhao, Joanna Lawrence, Michelle Towell, Heather D’Oyley, Marion Mackay-Dunn, Bryan Chow, Lillian Hung

**Affiliations:** 1 University of British Columbia Hospital—STAT Centre Inpatient, Vancouver Coastal Health, Vancouver, BC V6T 2B5, Canada; cromwellacosta@gmail.com (C.G.A.); joanna.lawrence@vch.ca (J.L.); michelle.towell@vch.ca (M.T.); marion.mackaydunn@vch.ca (M.M.-D.); bryan.chow@vch.ca (B.C.); 2 IDEA Lab, University of British Columbia, Vancouver, BC V6T 2B5, Canada; yye97@student.ubc.ca (Y.Y.); klywong1@student.ubc.ca (K.L.Y.W.); drzhao78@student.ubc.ca (Y.Z.); 3 School of Nursing, University of British Columbia, Vancouver, BC V6T 2B5, Canada

**Keywords:** bed sensors, sleep monitoring, implementation science, focus groups

## Abstract

Sleep is a crucial aspect of geriatric assessment for hospitalized older adults, and implementing AI-driven technology for sleep monitoring can significantly enhance the rehabilitation process. Sleepsense, an AI-driven sleep-tracking device, provides real-time data and insights, enabling healthcare professionals to tailor interventions and improve sleep quality. This study explores the perspectives of an interdisciplinary hospital team on implementing Sleepsense in geriatric hospital care. Using the interpretive description approach, we conducted focus groups with physicians, nurses, care aides, and an activity worker. The Consolidated Framework for Implementation Research (CFIR) informed our thematic analysis to identify barriers and facilitators to implementation. Among 27 healthcare staff, predominantly female (88.89%) and Asian (74.1%) and mostly aged 30–50 years, themes emerged that Sleepsense is perceived as a timesaving and data-driven tool that enhances patient monitoring and assessment. However, barriers such as resistance to change and concerns about trusting the device for patient comfort and safety were noted, while facilitators included training and staff engagement. The CFIR framework proved useful for analyzing implementation barriers and facilitators, suggesting future research should prioritize effective strategies for interdisciplinary team support to enhance innovation adoption and patient outcomes in rehabilitation settings.

## 1. Introduction

Sleep monitoring plays a crucial role in the overall well-being of older adults, offering insights into their health and quality of life [1]. As individuals age, their sleep patterns tend to change, often resulting in fragmented sleep, decreased total sleep time, and more frequent awakenings throughout the night [2]. Factors such as the hospital environment, including noise and light, can disrupt sleep patterns for older patients during their rehabilitation process. Additionally, medical conditions, medications, and anxiety related to hospitalization can contribute to poor sleep quality. We know older adults in hospitals experience a reduction in restorative sleep (rapid eye movement (REM) sleep and slow wave sleep) and frequent arousals and awakenings [3]. Poor sleep in hospitals for older adults can lead to an increased risk of delirium, slower recovery rates, and a higher likelihood of developing complications, including patient mortality. In contrast, improved sleep decreases the severity of muscle atrophy and weakness caused by sleep disturbance [4].

Monitoring sleep in older adults provides valuable information about the presence of sleep disorders such as insomnia, sleep apnea, and restless leg syndrome, which are prevalent in this population [5]. Identifying effective methods to assess sleep is essential as they can significantly impact physical health, cognitive function, and emotional well-being [6,7]. Inadequate sleep has been linked to an increased risk of chronic conditions, including cardiovascular disease, respiratory disease, stroke, diabetes, and cognitive decline in older adults [8]. Moreover, poor sleep quality can exacerbate existing health conditions and contribute to a decline in immune function, making older adults more susceptible to infections and illnesses [9]. By monitoring sleep patterns and identifying potential sleep disorders early on, healthcare professionals can implement targeted interventions such as cognitive-behavioral therapy for insomnia or continuous positive airway pressure (CPAP) therapy for sleep apnea, thereby improving sleep quality and overall health outcomes in older adults [10]. Additionally, tracking sleep metrics over time allows for the evaluation of treatment efficacy and adjustment of interventions as needed, ensuring optimal sleep health and enhancing the quality of life for older adults [11].

### 1.1. AI-Driven Sleep-Tracking Device and Sleepsense

AI-driven sleep-tracking sensors provide advanced monitoring and data analysis capabilities. Combining artificial intelligence and digital metrics, these sensors can track sleep patterns, support diagnosis, and assess the effectiveness of treatment [12]. By analyzing the collected data in real-time, AI systems can detect anomalies early, predict potential health issues, and alert healthcare providers to intervene promptly. This proactive approach enhances patient safety and care quality and contributes to personalized treatment plans for improving patient health outcomes. Adopting sensor technology in hospitals faces several challenges, including data interpretation, completeness and reliability, time constraints, technical issues, and patient data privacy concerns [13]. Healthcare providers have long relied on traditional observation and paper-based methods to track patients’ sleep; these methods can be labor-intensive and less accurate due to subjective observations. Yet, the adoption of sensor technology represents a shift in practice; acceptance among healthcare providers remains unknown. Despite wearable and non-wearable sleep-tracking devices becoming more common in healthcare [14,15], there is limited implementation research to understand staff acceptance and adoption in hospital settings.

The Sleepsense bed sensor is an AI-driven, non-wearable sleep-tracking sensor [16]. This palm-sized device utilizes passive sensing technology following the commonest form of the normal Ballistocardiogram (BCG) as posited by Albukhari et al. [17], in which BCG signals are extracted from the force transducer (load cell) [18]. These algorithmic technology solutions help create a nonobtrusive, non-invasive real-time sleep monitoring system wherein the bed sensor, encapsulated by an upper plate sensing surface and a stationary plate as its base, is manufactured with an accompanying electronic circuit with a built-in oscillator to induce that required frequency bandwidth [16]. This device is placed beneath one wheel or leg of the bed, resulting in the detection of signals from body movements either caused by cardiorespiratory activities or by any minute muscle or body movements, regardless of the subject’s position on the bed. Overall, Sleepsense bed sensors report essential health information such as sleep quality analysis and vital signs, particularly heart rate and respiratory rates. Sleep patterns are extrapolated from body movements, heart rate and respiratory rate. The application of this AI-driven sensor has been reported in Canadian long-term care (LTC) facilities, where it has increased successful transitions to LTC through data-driven evidence, enhanced residents’ sleep wellness by adjusting care plans based on sleep pattern data, and reduced falls through accurate bed-exit alerts [19].

Exploring perceived opinions about the acceptability of a new technology at the pre-implementation stage is critical for identifying potential barriers and designing tailored implementation strategies. Damschroder et al. [20] emphasize the importance of understanding interdisciplinary professionals’ perspectives to foster the successful implementation of health services research findings. Roger [21] and Greenhalgh et al. [22] highlight that those early perceptions of an innovation, such as its relative advantages, compatibility, and complexity, significantly impact its adoption and diffusion. Dearing and Cox [23] further suggest that understanding these perceptions can help overcome resistance and guide effective implementation strategies. Proctor et al. [24] argue that perceived opinions shape crucial outcomes like acceptability, feasibility, and appropriateness, which are vital for implementation success. Similarly, Venkatesh et al. [25] identify that factors influencing technology adoption, such as performance expectancy and social influence, are closely tied to perceived opinions. These perspectives collectively underscore the importance of assessing interdisciplinary staff attitudes and beliefs to support the effective adoption of new technologies or practices.

The purpose of this pre-implementation investigation is to explore the perceptions of interdisciplinary healthcare professionals regarding implementing an AI-driven bed sensor, Sleepsense, for monitoring patient sleep during the rehabilitation process in a geriatric unit, including their views on its potential benefits, challenges, and factors influencing its successful adoption into clinical practice. We wanted to know the facilitators and barriers to adopting Sleepsense; the results will be used to aid an implementation plan with tailored strategies to support adoption.

### 1.2. Research Questions

1. How do interdisciplinary healthcare professionals perceive the potential impact of introducing Sleepsense in a geriatric unit?
2. What will enable and hinder its adoption as a tool for patient care?

## 2. Methods

### 2.1. Design

Using the interpretive description approach [26], we conducted focus groups involving 27 staff members, including physicians, nurses, care aides, and other healthcare allies in a specialized older adult inpatient unit. This qualitative exploratory design focuses on gathering practical insights from staff to inform tailored strategies for adoption. Interpretive descriptions are well-suited for exploring the nuanced, complex experiences of staff in clinical contexts. This design allows us to yield practical, applied findings that can be used to refine the sensor’s design, improve training, and address specific concerns raised by staff, ensuring the technology is more acceptable and usable in real-world settings. The Consolidated Framework for Implementation Research (CFIR) informed our thematic analysis to identify barriers and facilitators to implementation [27]. CFIR provides a comprehensive approach to examine constructs such as innovation, inner and outer settings, and implementation processes, influencing technology adoption in hospitals and healthcare settings. The Focus group method was chosen as it allows for interactive discussions among participants, enabling the exploration of diverse viewpoints, experiences, and insights related to the research topic [28]. We report the results following the Consolidated Criteria for Reporting Qualitative Research (COREQ) (see Table S1 for the COREQ Checklist). The findings will be returned to Sleepsense developers for further improvement.

### 2.2. Sample

The study participants were healthcare practitioners working in a geriatric unit, including physicians, nurses, healthcare aids, and other healthcare allies involved in patient care. The geriatric unit is a sub-acute medical and psychiatric unit for older patients. This study utilized convenience sampling, providing an efficient and cost-effective way to gather data. The sample size provides confidence in effectively exploring the breadth and depth of our research questions. We put up posters and distributed flyers in staff conference rooms and unit walls. Participants were eligible to be involved in the research if they were (1) employed full-time or part-time, (2) directly involved in patient care, and (3) willing to participate in focus group discussions about Sleepsense adoption.

### 2.3. Data Collection

The focus groups were led by the Primary Investigator, an Asian male nurse, C.A., who holds an MSN (Master of Science in Nursing). C.A. conducted the focus groups via online meetings while participants were at the workplace. Before every focus group, a brief description of the sensor and how it operates were provided and the Tochtech Sleepsense link was shared with the participants to explore (https://www.tochtech.com/sleepsense-for-consumer (accessed on 21 October 2024)). The focus group lasted an average of 30 min and was audio-recorded and professionally transcribed verbatim. Field notes were also utilized during the group sessions to document non-verbal cues and other contextual factors that might have impacted the conversation. We used a focus group guide with three open-ended questions:

1. What are the benefits of integrating the Sleepsense into clinical practice?
2. What barriers do you anticipate in implementing Sleepsense technology in the geriatric unit, and how do you think these challenges could be addressed?
3. What are the key factors that would facilitate the successful adoption and integration of Sleepsense technology into the unit?

### 2.4. Data Analysis

Thematic analysis was utilized to identify patterns, themes, and insights concerning the viewpoints of interdisciplinary team members regarding the adoption of Sleepsense technology for monitoring patient sleep [29]. Our interdisciplinary team is composed of nurses (L.H., C.A. and Y.Y.), physicians (H.D., M.D., B.C. and Y.Z.), a social worker (K.W.), an occupational therapist (M.T.), and a physical therapist (J.L.). Y.Y. is an Asian female graduate in the Science of Nursing. Y.Z. is an Asian male health leadership and policy graduate with an M.D. degree. All team members engaged in independent repeated readings of the transcripts and field notes to familiarize themselves with all the data. Following this familiarization process, C.A., Y.Y., and Y.Z. coded data related to our research questions. Transcripts, quotations, and codes were managed using NVivo version 14.0. These codes were then discussed and organized into themes by C.A., Y.Y., and Y.Z. based on similarities and frequencies observed within the data. Throughout this process, themes were reviewed and refined with all research team members. Any discrepancies or alternative interpretations were addressed through team discussions until a consensus was reached. Upon establishment of the final set of themes and sub-themes, they were named and defined by C.A., Y.Y., and Y.Z. to reflect the data’s content accurately and subsequently interpreted in the context of the research questions. C.A., Y.Y., and Y.Z. wrote the first draft, and all authors were involved in making edits and agreed with the final draft. L.H. guided trainee authors throughout the data analysis process.

### 2.5. Ethical Considerations

The research was approved by the behavioral research ethics board of the University of British Columbia Office of Research Ethics (H23-01577). All participants provided a written consent form containing the research’s details, purpose, potential benefits, risks involved, and the right to withdraw. To ensure confidentiality, pseudonyms were used to anonymize participant identities.

### 2.6. Rigor

In this research, rigor is addressed through several methods. We engaged in reflective conversations at weekly research meetings and took reflexivity notes. Investigator triangulation was employed, with three diverse researchers making decisions regarding coding, analysis, and interpretations. Additionally, detailed documentation of the research, including data collection and analysis, was upheld to monitor the research progress and maintain an audit trail, fostering both accountability and transparency throughout the research process. To ensure the trustworthiness of our interpretation, we include direct quotations from interdisciplinary participants.

## 3. Results

In total, 27 staff from multiple disciplines who will participate in the next stage of implementing the Sleepsense sensor monitoring system accepted technological orientation and participated in the Sleepsense focus groups, including physicians, nurses, care aides, recreational staff and a pharmacist (Table 1). Female participants consisted of 88.89% of all participants. The majority of participants were between 30 and 50. Additionally, the participants were predominantly Asian (74.1%). All participants had worked in their positions for more than five years.

### 3.1. Theme 1: Timesaving and Data-Driven Tool

Nurses and care aides presume that this technology will be non-disruptive to patient sleep and will save time. They will no longer need to wake patients with sounds and flashlights to check if they are asleep. Sleep patterns are tracked automatically. They anticipate that this will improve efficiency in documentation, conserve time, and optimize resource allocation, leading to enhanced workflow and productivity overall. One care aide stated, “We will have less workload. You can see in the monitor. When a patient goes to the washroom, you know what room it is, and you can go to help” (Emma, care aide). Another person echoed, “This would reduce the need for so many night overtime” (Violet, care aide).

Participants valued the AI-driven technology’s ability to provide efficient and detailed data regarding patient sleep patterns. This function allows for more accurate diagnoses and personalized treatment plans, potentially improving the management of sleep disorders and optimizing patient outcomes. For example, a care aide remarked, “This is more accurate than subjective observation, which is our point of view, but the sensor is more like recording patient’s actual sleep experiences” (Amelia, care aide). A physician added, “It’s nice to know the detailed sleep pattern of the patient, with vital signs and sleep stages, trending, etc. By knowing the history of their night, our approach in the daytime can accommodate how they are feeling based on how they slept last night” (Lily, physician).

Sleep monitoring will enhance safety by preventing falls, improving mood, and supporting routine activities. Well-rested individuals are in better moods and are not fatigued, allowing more energy to engage in these activities. One staff member noted, “Sleep affects the patient’s behavior overall. If a patient lacks sleep, they would have irritability or agitation, making care more challenging” (Harper, nurse).

### 3.2. Theme 2: Barriers: Resistance to Change and Concerns About Trusting the Technology

Transitioning to Sleepsense involves a learning curve. One participant commented, “People rely on things they are comfortable with and familiar with. They are used to using it, so there would be a learning curve. Just like how we are transitioned from paper charts to electronic charting” (Hazel, physician). Some are worried about privacy and data security. A nurse expressed, “If something measures my sleep, I naturally feel anxious about it; I don’t know if I can trust it” (Aria, nurse). Other participants voiced several concerns about accessing and interpreting the data gathered by Sleepsense technology, coupled with a lack of familiarity with the technical aspects such as battery management. Participants also express worries about the perceived accuracy of the data collected by the sensor, especially in scenarios where patients do not sleep in standard positions. For example, a nurse asked, “If a patient sleeps on the side or in an unconventional position, not with their head or foot on the bed, how can Sleepsense accurately measure their sleep?” (Charlotte, nurse). Another person worried, “What if the tech is not working at night?” (Charlotte, nurse). A care aide added, “What if there is no power?” (Emma, care aide). “Money is important! Not sure if we have the budget for maintaining the technology!” (Violet, care aide).

### 3.3. Theme 3: Facilitators: Training and Staff Engagement

Orientation and training sessions are critical for the implementation of Sleepsense technology. Participants repeatedly underscored the importance of ongoing guidance and hands-on training sessions for incorporating and utilizing Sleepsense with current workflows. They also pointed to inclusive communication and training sessions for all team members, as well as opportunities for hands-on experience as key facilitating strategies. Many participants mentioned they were interested in learning data interpretations: “Training on how to access and make sense the data will surely help me to provide better care” (Sophia, physician). “We need some demo to train everyone so we can all master this technology” (Elizabeth, care aide). The focus group data demonstrated the need to prioritize theoretical and practical training to build confidence and competence to use Sleepsense effectively.

Engaging healthcare staff is vital for the successful adoption of Sleepsense technology. Staff involvement from the outset ensures that their insights and feedback shape the implementation process, addressing perceived concerns and fostering a sense of ownership. Participants were proud to have the AI-driven sensors for their geriatric unit and wanted to have the whole team involved in implementation. Participants emphasized that teamwork and excellent leadership are essential for overcoming challenges. “Everyone needs to be involved. So that we know how to troubleshoot and address minor problems that may arise, this communication and involvement of all the staff will facilitate the success of the project” (Emily, nurse).

Encouraging staff to share their perspectives with Sleepsense can enhance collective problem-solving and improve acceptance. For example, a care aide said, “Now I know it’ll ease off the workload, and it’ll give me more chance to pay attention to the patients who need me” (Eleanor, care aide). “I am hoping for more reliable data that is less human error. And I think there’d be more reliability of reporting” (Hazel, physician).

## 4. Discussion

This study explored the perspectives of an interdisciplinary hospital team on implementing the AI-driven sleep-tracking device Sleepsense in geriatric hospital care. The findings on anticipated impact and concerns are aligned with the results of other studies. For example, Ravichandran et al. [30] reported that sleep sensor technology enhances awareness of sleep health and facilitates healthy sleep habits; traditional sleep monitoring methods by staff observation can disturb sleep. Bandyopadhyay et al. [31] highlighted several barriers encountered when applying AI-enabled technology in sleep medicine, including a lack of knowledge about AI algorithms, patient privacy concerns, and accessibility issues. In contrast, wearable devices may increase sleep cycle fragmentation and disrupt circadian rhythms in older adults with dementia [32]. The physical presence of the device can be uncomfortable or intrusive, leading to increased movement and wakefulness during the night. Additionally, the light and notifications from these devices can disturb sleep. For individuals with dementia, who are already vulnerable to sleep disturbances and circadian rhythm disruptions, these factors can exacerbate their condition. The cognitive load of understanding and adapting to a new device can also be stressful, further impacting sleep quality and stability. Bed sensors offer the least disruptive and comfortable user experience [33].

Our study’s findings were informed by the CFIR, which provides a comprehensive approach to understanding the various factors that influence the implementation of innovations in healthcare settings. The qualitative analysis of staff responses in focus groups highlighted alignment with several CFIR constructs (e.g., the relative advantages of the innovation, team culture, knowledge and training, and engagement) that can play critical roles in the successful adoption of sleep tracking sensors. For example, staff reported time-saving as one of the relative advantages of the Sleepsense technology. They also valued the device as a helpful data-driven tool to support patient care. Other CFIR constructs, such as staff engagement in implementation planning, compatibility with workflows, and team culture, were also found across focus group data.

Active staff engagement in the planning and implementation of Sleepsense was a key facilitator. By involving staff from the outset, their insights and feedback can be incorporated to help address concerns and foster a sense of ownership. This engagement is crucial as it builds commitment and ensures that the staff feels valued and invested in the successful adoption of the technology. The compatibility of Sleepsense technology with existing workflows was another significant factor. The technology’s ability to integrate into current practices can make it more acceptable to staff. For example, the automatic tracking of sleep patterns without disturbing patients fits well with the need for non-intrusive and efficient monitoring, which enhances workflow and productivity. The intervention’s alignment with the team culture also contributes to its acceptance. The collaborative approach resonated well with the staff, emphasizing inclusive team training and communication. Participants repeatedly underscored the importance of collective problem-solving and shared responsibility in overcoming challenges associated with the new technology. Our results suggest that focusing on these CFIR constructs—staff engagement, compatibility with workflows, and alignment with inclusive team culture—will likely contribute to the successful integration of Sleepsense technology in geriatric hospital care. The insights gained from this study can inform future implementation of AI-driven technology aimed at enhancing patient care during the rehabilitation process, ensuring that they are both effective and well-received by the healthcare teams responsible for their use.

Using multiple approaches to assess sleep is essential to understand sleep patterns and issues comprehensively. Combining objective measures, such as sleep-tracking devices, with subjective methods, like patient-reported sleep experience, provides a fuller picture of sleep health. This multifaceted assessment allows for data cross-validation, enhancing the findings’ reliability. Other sleep recording approaches in clinical settings, such as clinical observations and patient perceptions, can provide complementary perspectives on sleep monitoring. The most acceptable tools are the Sleep Observation Tool (SOT) for clinician observations and the Richard Campbell Sleep Questionnaire (RCSQ), respectively [34]. However, studies show little correlation between clinician-based assessments and patient self-reports due to their different focuses [35]. While patients often report difficulties falling asleep and the quality of their sleep, clinicians tend to focus on the integrity of the sleep–wake cycle. Objective assessment methods using an AI-driven sensor can help to address the difference between clinicians’ observations and patients’ experiences, bridging the gap between clinician and patient viewpoints [36]. Clinician observation typically depends on certain indicators: eyes being closed, minimal interaction with the environment, reduced motor activity, and an absence of deliberate actions, all of which can compromise their assessments of actual sleep [37], a concern consistently echoed by our frontline staff participants.

Including patient experiences in sleep assessment and management is crucial. Understanding patients’ subjective sleep experiences and perceptions of different sleep-tracking technologies can provide valuable insights that enhance the overall quality of care. This approach ensures that interventions are evidence-based and tailored to patients’ individual needs and preferences, thereby improving adherence and outcomes. Engaging patients in their care process fosters a collaborative environment and empowers them to actively manage their sleep health. Patients’ subjective experiences, including how rested they feel or their perceived sleep disturbances, add valuable context to sleep assessments. Self-reported sleep quality is strongly related to chronic diseases, poor mental health, and increased suicide risk [38]. Even with advanced sleep monitoring technologies, patient self-reports remain crucial. They should be included in sleep assessment tools as long as patients are alert, oriented, and able to provide feedback [37]. Beyond the value of patient self-reports, clinical observations provide valuable information but can also disrupt sleep. Various monitoring and recording approaches can alter patients’ perceptions of their sleep [33]. For example, polysomnography (PSG) can accurately record sleep parameters but may cause severe discomfort, failing to reflect actual, uninterrupted sleep. A similar situation occurs when frontline staff record sleep. Instead of the discomfort brought by wearable sensors, frontline staff often directly interrupt patients’ sleep. As reported in our focus groups, patients notice sleep checks happening every half hour, causing them to wait for these regular visits. The noise and flashlight from these around-the-clock checks further disturb their rest. This is why frontline staff express a strong preference for unobtrusive technologies.

Implementing AI-driven sleep monitoring devices, such as Sleepsense bed sensors, as an innovative technology requires knowledge translation (KT), which demands education and training. Knowledge translators need to tailor key information into individualized formats or languages according to different target audiences for better acceptance [39]. This includes addressing user acceptance and training challenges, as resistance to new technology and inadequate training are common barriers to successful adoption. Training programs must ensure that participants are familiar with the operation of Sleepsense, including its setup, data interpretation, and troubleshooting procedures. Building confidence and technical proficiency through hands-on training is critical to improving the acceptance and usability of the system. Additionally, reliability and maintenance concerns, such as ensuring the system functions during power outages or technical failures, must be addressed through robust support and backup systems. The wide range of stakeholders involved in implementing sleep monitoring technologies necessitates interdisciplinary research. Our study includes researchers, physicians, nurses, care aides, recreational staff, and a pharmacist. In the next step, we plan to involve patients, managers, occupational therapists, and physical therapists in evaluating the process of implementation. Successful KT among healthcare professionals relies on a thorough assessment of the likely barriers and facilitators all stakeholders face [39].

Some staff raised concerns about consent and the privacy and security of patient information. The IoT paradigm, with advances in sensing devices, cloud and fog resources, and Big Data technologies, provides a robust framework for pervasive health monitoring [40]. While this offers opportunities to enhance hospital information systems, it also introduces privacy risks. Storing data in fog resources can mitigate some of these risks [33], and encryption enhances data privacy, though it complicates processing. Fog technology, offering localized data storage, has advantages over cloud-centralized networks [41]. Focus groups revealed concerns about data confidentiality, particularly as many patients may not fully understand the consent process for bed sensors. Before deploying Sleepsense, it is essential to ensure that patient data are securely collected, analyzed, and stored, safeguarding privacy whether using cloud, fog resources, or Big Data technologies.

Most new digital developments encounter numerous barriers inherent to the medical sector, with resistance to change being particularly significant [42]. Sources of resistance during the implementation stage include value conflicts, departmental politics, uncertainty, leadership inaction, embedded routines, lack of coordination, lack of capabilities, and cynicism [42]. Bed sensors, aimed at improving efficiency, help mitigate value conflicts and departmental politics. Implementing bed sensor-based sleep monitoring will involve multiple training sessions from installation to operation to address the technical gaps frontline staff face. However, technician support will still be required for maintenance, presenting slight barriers to implementation. While the introduction of bed sensors changes well-established practical routines, it is not expected to evoke negative emotions due to the time-saving and user-friendly nature of the technology. The deployment process engages all stakeholders from the beginning, emphasizing coordination, teamwork, and trust, which helps reduce reluctance to change. The challenge to overcome barriers depends on the new technology’s disruption level and the number of variable conventional procedures [43]. Currently, somnolog is the sole method for sleep monitoring in the geriatric unit. The absence of other conventional procedures and the relatively low complexity of bed sensor technology will result in the lowest level of barriers.

The bi-directional relationship between sleep disorders and serious medical problems like hypertension, depression, cardiovascular diseases, and cerebrovascular disease is often underappreciated and under-addressed in clinical practice [11]. Patients experiencing sleep disorders are affected by three main factors—emotional or physical impairment due to illness or hospitalization, sleep disturbance due to care plan schedules, and sleep interruption due to the hospital environment or medical care—which highlights the need for improvements in clinical environments and modifications of medical and care plans [44]. Identifying potential sleep disorders and providing evidence-based modifications to medical and care plans relies on the routine monitoring of sleep patterns and vital signs. Bed sensors for sleep monitoring have emerged as valuable tools in enhancing sleep medicine and care plans [33]. These sensors provide a comprehensive picture of sleep patterns and quality by tracking various physiological parameters, including heart rate, respiratory rate, movement, and sleep stages. This ensures early detection of sleep disorders such as sleep apnea, restless legs syndrome, and insomnia, enabling timely intervention and management by physicians [11]. Healthcare providers can use data from bed sensors to tailor treatments and interventions based on individual sleep patterns and needs. Frontline staff in this study also expressed a strong demand for sleep monitoring approaches during clinical treatment and care, and they emphasized the need for more accurate and continuous sleep monitoring technologies. This supports the integration of new bed sensors into current clinical applications, rationalizing their acceptance and utility in improving patient care.

Our findings provide important implications for future research and practice. Implementing Sleepsense is anticipated to potentially improve sleep assessments, reduce the workload of healthcare staff, and enhance patient care by providing sleep details and histories that inform medication management and care planning. Training and orientation sessions are crucial for successful implementation, ensuring that healthcare professionals are familiar with the technology and can effectively incorporate it into their workflows. AI-driven measurement in gerontological practice can inspire the interest and curiosity of frontline staff. Evidence still needs to be built to address the concerns of frontline staff to build trust and acceptance of AI-driven technology. Orientation and training, peer cooperation, and leadership support could facilitate the implementation of innovative technology in healthcare settings. Future research should focus on several key areas to advance sleep assessment and management. First, studies should evaluate the long-term effects of various sleep-tracking technologies on sleep quality and overall health, especially in older adults and those with dementia. Additionally, research should explore the development of more user-friendly and less intrusive devices that minimize sleep disruption. Investigating the integration of multimodal assessment tools, including subjective and objective measures, can enhance the accuracy and comprehensiveness of sleep evaluations. Furthermore, future studies should prioritize the inclusion of diverse patient populations to ensure that findings are generalizable and applicable across different demographic groups. Future research can contribute to more effective and personalized sleep health by addressing these areas.

### Limitations

This study has several limitations. Firstly, the sample consisted of only 27 participants, with the majority being healthcare aides. While efforts were made to include diverse perspectives, this limited sample size may not fully capture the diverse viewpoints of all healthcare professionals who could utilize Sleepsense technology. Therefore, this finding may not be fully representative of the broader population of healthcare professionals. Additionally, this study was conducted within a specific geriatric hospital unit. The findings may not be generalizable to other healthcare settings with different patient populations.

Furthermore, participants’ responses during interviews influenced the possibility of social desirability bias. Participants may have provided answers that they believed were expected by the researchers, potentially influencing the accuracy of the data. Moreover, constraints in funding may have limited the depth of the study by restricting the scope of research and the ability to conduct longitudinal follow-ups, preventing a comprehensive exploration of all relevant factors influencing Sleepsense implementation. Future research could further explore the multifaceted perspectives of healthcare professionals regarding factors influencing Sleepsense implementation.

## 5. Conclusions

In conclusion, this study highlights the perceived benefits and challenges of implementing Sleepsense technology in healthcare settings. While significant interest and recognition of its advantages exist, addressing concerns, providing adequate support, and training are essential for successful adoption. Future research and practice efforts should focus on creating an environment conducive to technological innovation, ensuring that healthcare professionals have an active voice and are engaged in implementing AI-driven innovations. Staff viewed the sleep sensor as a valuable tool for improving patient care. They appreciated that the sensor is non-disruptive to patient sleep, which is critical for maintaining patient well-being and safety. The staff recognized that the sensor could save time, enhance patient sleep quality, help prevent falls, improve mood, and support patients in their routine activities. However, they also expressed concerns regarding the need for adequate training and addressing potential privacy issues. To ensure successful adoption, these concerns should be addressed by involving staff in the planning and implementation process, fostering engagement, and providing the necessary training for adoption.

## Figures and Tables

**Table 1:** Demographic characteristics of the participants.

Demographic Characteristic	n	%
Role		
Physician	6	22.22
Pharmacist	1	3.70
Nurse	11	40.74
Care aide	8	29.63
Recreational staff	1	3.70
Gender		
Male	3	11.11
Female	24	88.89
Age group		
30–40 years	8	29.63
40–50 years	10	37.04
50–60 years	3	11.11
60–70 years	6	22.22
Ethnicity		
Asian	20	74.1
White	5	18.5
African Canadian	2	7.4
Working experience		
5–10 years	8	29.63
10–20 years	12	44.44
20–30 years	7	25.93
Total	27	

## Data Availability

We do not have any research data outside the submitted manuscript file.

## Supplementary Materials

The following supporting information can be downloaded at: https://www.mdpi.com/article/10.3390/s24216803/s1, Table S1: COREQ Checklist. Reference [45] is cited in the Supplementary Materials.
