# Supplementary material for: Implementing AI-Driven Bed Sensors: Perspectives from Interdisciplinary Teams in Geriatric Care
Source: Sensors (Basel). 2024 Oct 23;24(21):6803. doi: 10.3390/s24216803 (PMC11548467; doi:10.3390/s24216803)
Supplement: Supplementary file 1 [file sensors-24-06803-s001.zip › sensors-3148811-supplementary.pdf]

**Table S1.** Consolidated Criteria for Reporting Qualitative Studies (COREQ): 32-item Checklist

| No. Item | Guide questions/description | Reported on Page # |
|---|---|---|
| Domain 1: Research team and reflexivity | | |
| Personal Characteristics | | |
| 1. Interviewer/facilitator | Which author/s conducted the interview or focus group? | Page 3 |
| 2. Credentials | What were the researcher's credentials? e.g., PhD, MD | Page 3 |
| 3. Occupation | What was their occupation at the time of the study? | Page 3 |
| 4. Gender | Was the researcher male or female? | Page 3 |
| 5. Experience and training | What experience or training did the researcher have? | Page 3 |
| Relationship with participants | | |
| 6. Relationship established | Was a relationship established prior to study commencement? | Page 4 . |
| 7. Participant knowledge of the interviewer | What did the participants know about the researcher? e.g., personal goals, reasons for doing the research | Page 4 |
| 8. Interviewer characteristics | What characteristics were reported about the inter viewer/facilitator? e.g., Bias, assumptions, reasons and interests in the research topic | Page 4 |
| Domain 2: study design | | |
| Theoretical framework | | |
| 9. Methodological orientation and Theory | What methodological orientation was stated to underpin the study? e.g., grounded theory, discourse analysis, ethnography, phenomenology, content analysis | Page 3 |
| Participant selection | | |
| 10. Sampling | How were participants selected? e.g., purposive, convenience, consecutive, snowball | Page 3 |
| 11. Method of approach | How were participants approached? e.g., face-to-face, telephone, mail, email | Page 3 |
| 12. Sample size | How many participants were in the study? | Page 3 |
| 13. Non-participation | How many people refused to participate or dropped out? Reasons? | NA |
| Setting | | |

| | | |
|---|---|---|
| 14. Setting of data collection | Where was the data collected? e.g., home, clinic, workplace | Page 3 |
| 15. Presence of non-participants | Was anyone else present besides the participants and researchers? | Page 3 |
| 16. Description of sample | What are the important characteristics of the sample? e.g., demographic data, date | Page 4 |
| Data collection | | |
| 17. Interview guide | Were questions, prompts, guides provided by the authors? Was it pilot tested? | page 3 |
| 18. Repeat interviews | Were repeat interviews carried out? If yes, how many? | NA |
| 19. Audio/visual recording | Did the research use audio or visual recording to collect the data? | Page 3 |
| 20. Field notes | Were field notes made during and/or after the interview or focus group? | Page 3 |
| 21. Duration | What was the duration of the interviews or focus group? | Page 3 |
| 22. Data saturation | Was data saturation discussed? | NA |
| 23. Transcripts returned | Were transcripts returned to participants for comment and/or correction? | NA |
| Domain 3: analysis and findings | | |
| Data analysis | | |
| 24. Number of data coders | How many data coders coded the data? | Page 3 |
| 25. Description of the coding tree | Did authors provide a description of the coding tree? | Page 5 & 6 |
| 26. Derivation of themes | Were themes identified in advance or derived from the data? | Page 5 & 6 |
| 27. Software | What software, if applicable, was used to manage the data? | Page 3 |
| 28. Participant checking | Did participants provide feedback on the findings? | NA |
| Reporting | | |
| 29. Quotations presented | Were participant quotations presented to illustrate the themes/findings? Was each quotation identified? e.g., participant number | Page 5 & 6 |
| 30. Data and findings consistent | Was there consistency between the data presented and the findings? | Page 5 & 6 |
| 31. Clarity of major themes | Were major themes clearly presented in the findings? | Page 5 & 6 |

| | | |
|---|---|---|
| 32. Clarity of minor themes | Is there a description of diverse cases or discussion of minor themes? | Page 5 & 6 |

Note. NA = not applicable.

Developed from:

Tong, A.; Sainsbury, P.; & Craig, J. Consolidated criteria for reporting qualitative research (COREQ): A 32-item checklist for interviews and focus groups. *International Journal for Quality in Health Care* **2007**, *19(6)*, 349-357, doi:10.1093/intqhc/mzm042
